# A Scoping Review of Technology-Based Approaches for Upper Limb Motor Rehabilitation after Stroke: Are We Really Targeting Severe Impairment?

**DOI:** 10.3390/jcm13185414

**Published:** 2024-09-12

**Authors:** Emma Colamarino, Giovanni Morone, Jlenia Toppi, Angela Riccio, Febo Cincotti, Donatella Mattia, Floriana Pichiorri

**Affiliations:** 1 Department of Computer, Control, and Management Engineering “Antonio Ruberti”, Sapienza University of Rome, 00185 Rome, Italy; emma.colamarino@uniroma1.it (E.C.); jlenia.toppi@uniroma1.it (J.T.); cincotti@diag.uniroma1.it (F.C.); 2 IRCCS Fondazione Santa Lucia, 00179 Rome, Italy; a.riccio@hsantalucia.it (A.R.); d.mattia@hsantalucia.it (D.M.); f.pichiorri@hsantalucia.it (F.P.); 3 Department of Life, Health and Environmental Sciences, University of L’Aquila, 67100 L’Aquila, Italy

**Keywords:** technological interventions, motor rehabilitation, upper limb, stroke, severe impairment

## Abstract

Technology-based approaches for upper limb (UL) motor rehabilitation after stroke are mostly designed for severely affected patients to increase their recovery chances. However, the available randomized controlled trials (RCTs) focused on the efficacy of technology-based interventions often include patients with a wide range of motor impairment. This scoping review aims at overviewing the actual severity of stroke patients enrolled in RCTs that claim to specifically address UL severe motor impairment. The literature search was conducted on the Scopus and PubMed databases and included articles from 2008 to May 2024, specifically RCTs investigating the impact of technology-based interventions on UL motor functional recovery after stroke. Forty-eight studies were selected. They showed that, upon patients’ enrollment, the values of the UL Fugl-Meyer Assessment and Action Research Arm Test covered the whole range of both scales, thus revealing the non-selective inclusion of severely impaired patients. Heterogeneity in terms of numerosity, characteristics of enrolled patients, trial design, implementation, and reporting was present across the studies. No clear difference in the severity of the included patients according to the intervention type was found. Patient stratification upon enrollment is crucial to best direct resources to those patients who will benefit the most from a given technology-assisted approach (personalized rehabilitation).

## 1. Introduction

Most technology-based approaches for motor rehabilitation after stroke have been originally designed and developed with severely affected patients in mind [1,2]. In post-stroke rehabilitation, traditional approaches targeting upper limb function often rely on the presence of residual motor capabilities and are, therefore, precluded to very severe (i.e., plegic) patients. Furthermore, technology can provide treatment with increased intensity, which is established as a crucial determinant of rehabilitation outcome [3]. In this regard, Constraint-Induced Movement Therapy (CIMT), which is still one of the most effective approaches for the upper limbs [4], represents a clear example of how technology can impact motor recovery only in those patients with residual motor function of the affected limb. The lack or scarceness of such residual motor function can be, at least partially, overcome with technology, such as neuromuscular stimulation combined with CIMT [5]. Thus, technology can not only enhance the intensity of the treatment but also increase the number of patients that can have access to a given therapy, increasing the chances for all patients (including those more severely affected) to regain independence.

However, randomized controlled trials (RCT) testing these technology-based rehabilitation approaches often include patients with a wide range of motor impairment and, sometimes, only a small proportion of patients actually fall in the severe range as defined by clinical scales. Indeed, patients’ recruitment in rehabilitation is influenced by numerous factors and is still one of the most challenging steps for researchers aiming to bring technology into clinical practice [6]. The severity of motor deficit often comes with concomitant conditions, such as bed-confinement status (especially in the acute and subacute phases), cognitive impairment, pain, and depression, that altogether limit the ability of patients to participate in such trials. As the motor status at baseline is probably the most consistent prognostic factor of rehabilitation outcome [7], we believe that this factor accounts for the hesitating translational success of many of these technological approaches for several reasons. On one hand, the success of clinical trials may be facilitated by the participation of less severe patients who increase trial feasibility, especially in the subacute phase, and are naturally destined to a more favorable outcome. Consequently, the actual application of a given technology in severe patients may turn out disappointing in terms of outcome, regardless of the successful trial. On the other hand, some of these technological aids may provide little added benefit for less severe patients who can take advantage of other more traditional and less expensive rehabilitative exercises [4]. Furthermore, several aspects related to patients’ recruitment add complexity to the ambitious task of proving the efficacy of novel rehabilitation strategies, such as post-stroke stage and the related adaptive and maladaptive changes that occur both at the central nervous system level and in peripheral tissues (e.g., spasticity).

In this scoping review, we provide an overview on the actual severity of patients recruited in RCTs carried out to analyze the efficacy of technology-based rehabilitation approaches targeting severe upper limb impairment (explicitly mentioning patients’ severity in the title and/or abstract). According to our question, our research returned clinical studies investigating robotics, electrical or magnetic stimulation of the central nervous system (non-invasive brain stimulation, NIBS) or of peripheral structures (peripheral stimulation, PS), brain–computer interfaces (BCIs), virtual reality (VR), and, in general, advanced technological devices purposely developed for post-stroke motor rehabilitation. While the identification of the most effective approaches is out of the scope of this review [8], here we intend to verify the following:

(i) The actual severity of patients included in trials that explicitly declare to enroll severe subjects to confirm or refute the anecdotal notion of extreme variability in baseline motor impairment, which might be responsible for the hesitating translational success of such interventions [9];
(ii) Whether some of these technological approaches have been more consistently tested on severe patients than others and eventually speculate on why they have.

Furthermore, we check whether the severity of patients was considered as an inclusion criterion and/or employed for stratification for primary or secondary analyses. We take into account whether each considered study was or was not successful in confirming the efficacy of a given intervention, that is, the superiority/not superiority of the target intervention vs. the control condition (positive/negative results), also highlighting possible secondary analyses carried out by the authors to support their hypotheses.

The ultimate goal of this scoping review is to provide useful hints to improve patients’ inclusion in RCTs evaluating novel technologies for motor rehabilitation in order to favor the optimization of resources and efforts towards tailored, highly technological rehabilitation interventions, which is instrumental to foster technology transfer into clinical practice.

## 2. Methods

### 2.1. Protocol and Eligibility Criteria

This scoping review was conducted according to the Preferred Reporting Items for Systematic reviews and Meta-Analysis (PRISMA) standards. Our protocol was drafted using the PRISMA extension for Scoping Reviews (PRISMA—ScR, [10]) and revised by the research team. To be included in the review, papers needed to describe technology-based rehabilitative interventions targeting the recovery of motor function of the upper limb in a stroke population.

Peer-reviewed journal papers were included as follows:

- Published between the period of 2008 and 2024;
- Written in English;
- Involved human participants in the framework of a randomized controlled trial.

Papers were excluded as follows:

- If they did not fit into the conceptual framework of the study (not a technology-based rehabilitative approach, wrong outcome);
- If they were reviews, study protocols, and meta-analyses.

### 2.2. Information Sources and Search Strategy

Studies eligible for review were identified through electronic databases, such as Scopus and PubMed, from 2008 to 23 May 2024. Grey literature was excluded from the search process. The search strategies consisted of free text terms in the topic “stroke” AND “severe” AND “rehabilitation” AND “Action Research Arm Test” OR “Fugl-Meyer Assessment” AND “randomised controlled trial” AND “upper limb” OR “hand”. The complete search terms and strategy are provided in the Supplementary Materials (Table S1). The search strategies were developed and executed by a biomedical engineer (EC) and further refined through team discussion. The search was peer-reviewed by other expert researchers, i.e., a neurologist (FP) and a physiatrist (GM), using the Peer Review of Electronic Search Strategies checklist and modified as required [11]. The final search results were imported into the online systematic review software Rayyan [12]. Duplicates were identified by means of the Rayyan duplicates search algorithm and removed by a researcher.

### 2.3. Selection of Sources of Evidence

Two reviewers (EC, FP) independently screened titles and abstracts for inclusion. For full-text screening, two reviewers (EC, FP) subsequently screened the full text of potentially relevant articles to determine inclusion using similar inclusion and exclusion criteria. We resolved disagreements on study selection by consensus and discussion with other reviewers if needed or by a single arbitrator (GM). To ensure reliability between reviewers, a series of training exercises were conducted prior to commencing screening. Inter-rater agreement for study inclusion was calculated using percent agreement; when it reached >80% across the team, we proceeded to the next stage. If lower agreement was observed, the inclusion and exclusion criteria were clarified, and another pilot test occurred.

### 2.4. Data Charting Process and Data Items

A data charting form was jointly developed by three reviewers to determine which variables to extract. Three reviewers (EC, FP, GM) independently charted the data, discussed the results, and continuously updated the data charting form in an iterative process.

For each article, data on the following characteristics were extracted:

- First Author Name;
- Year of publication;
- Source;
- Population sample size (participants per group);
- Severity of the upper limb impairment, i.e., Upper Extremity Fugl-Meyer Assessment score, FMA [13], and/or Action Research Arm Test, ARAT [14], expressed as the mean ± standard deviation (SD) or median and first and third quartile (Q1–Q3), per group, whenever available;
- Inclusion criteria in the RCT related to the upper limb impairment;
- Availability of the dataset used (Yes/No);
- Time since injury (TSI), i.e., stroke event, classified, according to [15,16], as
  ○ ≤1 month (acute)
  ○ ≤3 months (early subacute)
  ○ ≤6 months (subacute)
  ○ >6 months (chronic)
- Intervention type, classified as
  ○ Brain–Computer Interface (BCI)
  ○ Non-Invasive Brain Stimulation (NIBS)
  ○ Peripheral Stimulation (PS)
  ○ Robotic
  ○ Virtual Reality (VR) and Visual
- Comparator, i.e., control interventions and/or comparison conditions;
- Active motor action required (Yes/Yes whenever possible/No):
  ○ *Yes*, if the intervention type requires the participant’s residual motor ability (active motor exercise from the participant)
  ○ *Yes whenever possible* refers to conditions foreseeing active motor exercise when feasible, with the technology providing assistance as needed (e.g., robotics)
  ○ *No* otherwise
- Combination of technological interventions (Yes/No);
- Dose, expressed as minutes x number of sessions;
- Primary and secondary outcomes;
- Key Findings, classified as Positive, Positive on secondary analyses, and Negative. We define Key Findings as the following:
  ○ *Positive* if between-group statistical analyses evaluated for the primary outcomes statistically confirm the hypothesis investigated in the study.
  ○ *Positive on secondary analyses* if between-group statistical analyses evaluated for sub-items of the primary/secondary outcomes or considering sub-groups of the population under investigation confirm the hypothesis investigated in the study or if within-group statistical analyses evaluated for the primary/secondary outcomes reveal a statistical improvement only for the experimental group.
  ○ *Negative* if between- and within-group analyses do not reveal statistically significant differences among groups.

The threshold for statistical significance was set to 0.05.

- Stratification for secondary analyses according to an upper limb impairment criterion;
- Follow-up (Yes/No), i.e., if *Yes*, we reported the number of months after the end of the intervention;
- Setting: Inpatient/Outpatient.

The extracted data were collected in a table in which the rows represent the included articles and the columns represent variables (data items). The spreadsheet software Microsoft Excel (Version 2408) was used to create our custom extraction form. The choice was based on its ease of use, high customizability, and worldwide diffusion. Before extracting the data from all papers included in the scoping review, the extraction form was tested for further refinements and underwent a calibration phase. This entailed three reviewers independently extracting data from 5 papers each and meeting afterward to discuss any discrepancies, with further refinement of the form if a high level of agreement between reviewers was not obtained.

### 2.5. Synthesis of Results

To analyze the database, the following explanatory approaches are used: descriptive and frequency analysis and association analysis.

#### 2.5.1. Descriptive and Frequency Analysis

Descriptive statistics are relative to the overall population of participants that was included in the selected studies in terms of the number of participants, sample size of intervention and control groups, dose of intervention, and severity of upper limb motor impairment upon enrollment as described by FMA and/or ARAT. The data have been summarized according to their distribution (modality and dispersion) by means of the mean and standard deviation (SD) or median and interquartile range (IQR), presented as the difference between the first quartile (Q1) and the third quartile (Q3), i.e., Q1–Q3.

Frequency analyses are relative to the following variables: time since injury (at least 4 classes, i.e., acute, early subacute, subacute, chronic participants), setting (at least 3 classes, i.e., inpatient/outpatient, inpatient, and outpatient), availability of the dataset used (2 classes, i.e., Yes or No), technological rehabilitative intervention type (at least 5 classes, i.e., BCI, NIBS, PS, Robotic, VR and Visual) and whether it did or did not require an active motor exercise from the participants (3 classes, i.e., Yes/Yes whenever possible/No), comparison conditions, primary and secondary outcome measures, presence/absence of follow-up evaluations (2 classes, i.e., Yes or No), key findings (3 classes, i.e., positive, negative, and positive on secondary analyses), and severity of upper limb deficit employed as an inclusion criterion for participant enrollment and/or stratification for secondary statistical analyses. In frequency analysis, the counts and percentages of articles in each cluster are calculated. Studies that share a similar approach towards a specific variable are clustered together, and those following different approaches are assigned to different groups. Clustering can be carried out based on the values of a single variable on the entire dataset or on a subset of articles that already belong to a cluster on a higher level.

All results are presented both narratively and by means of plot and pie charts when relevant.

#### 2.5.2. Association Analysis

Association analysis explores the relationships between the variables. Since the number of possible combinations of variables is relatively large, the results are focused on those regarding the research questions. We hypothesize that the characteristics of the technological rehabilitation interventions would determine a difficulty in recruiting severely affected participants. Therefore, we have separately analyzed the severity of upper limb motor impairment at baseline (as assessed via FMA) according to the following:

- Type of intervention (e.g., Robotic, BCI, PS, …);
- Required active upper limb motor actions from the participant by the intervention itself.

For each analysis, studies that share a similar approach, i.e., type of intervention or required active motor action, are clustered together. For each level of the analyzed variable, i.e., 5 levels for the variable TYPE OF INTERVENTION and 3 levels for the variable MOTOR ACTION, the FMA data are pooled together. If needed, the mean and SD data are estimated from the data reported in the paper as the median and IQR by means of the formula in [17].

All results are expressed as the mean ± SD and presented both narratively and by means of plot charts.

## 3. Results

### 3.1. Selection of Sources of Evidence

The search returned a total of 189 papers. After the duplicate removal (96), 93 articles were screened. After screening of titles and abstracts, 41 papers were excluded due to the following reasons:

- The rehabilitative intervention under investigation does not include a technology-based approach; videos/instruction displayed on screens or other devices of everyday use (personal computers, tablet, smartphones) were not included;
- The design of the study does not follow the randomized controlled trial design (wrong study design);
- The effectiveness of the rehabilitative intervention under investigation was not assessed in terms of motor function improvement (wrong outcome);
- The paper presents a study protocol, a review, or a meta-analysis.

A total of 52 full-text papers were examined. Four studies were excluded during the full-text search and check. Thus, 48 articles were included in the scoping review.

**Figure 1:**
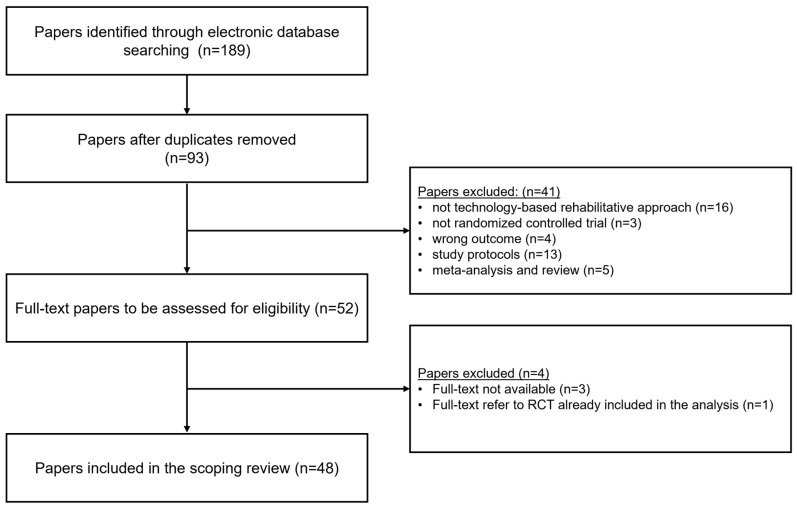
Flowchart of search and selection process.

The flowchart in Figure 1 presents the detailed search and selection process.

### 3.2. Results of Individual Sources of Evidence

The results of individual sources of evidence are shown in Table 1. Table 1 reports a subset of the items described in the section *Data charting process and data items*.

### 3.3. Synthesis of Results

#### 3.3.1. Descriptive and Frequency Results

The included articles report data from 3000 adult participants. Three articles [20,25,54] include participants with a diagnosis of stroke and traumatic brain injury (TBI), respectively five and three TBI participants in [20] and [25], for a total of eight participants. No detailed information on stroke/TBI ratio is reported in [54].

The average sample size of the groups (target intervention and control groups) is 29 ± 39 (mean ± SD). The RCT in [41] is the only study in which a very large number of participants, i.e., 770 participants, were enrolled. Conversely, there is more than one study in which a very small number of participants, i.e., less than 10 per group, are analyzed [23,29,36,46,47,51,60]. Therefore, median and quartile values, i.e., 20 (median) and 11–32 (Q1–Q3), provide more accurate estimates on the target intervention and control groups sample size. Among the included studies, only 45.83% [18,19,22,25,26,27,30,32,33,34,35,38,41,45,46,49,50,53,58,59,62,63] performed a sample size calculation, and 81.82% [18,22,25,26,30,32,33,34,35,38,41,46,49,50,53,59,62,63] of them actually enrolled the foreseen number of patients.

**Figure 2:**
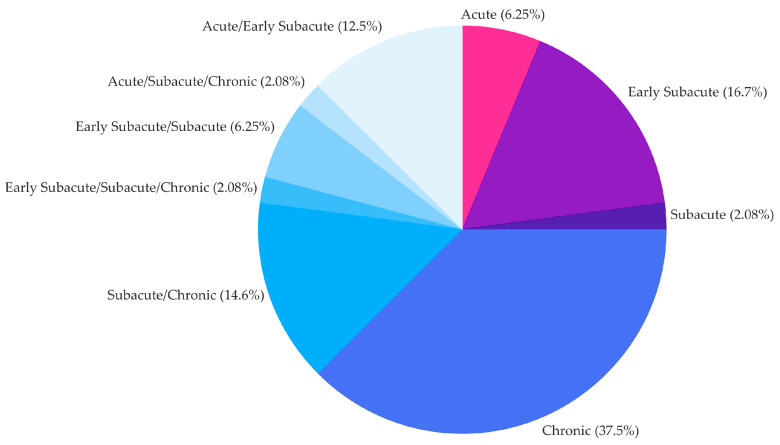
Distribution across studies (*n* = 48) of the stroke population enrolled and classified in terms of time from the cerebral lesion to enrollment in the study as follows: ≤1 month (acute), ≤3 months (early subacute), ≤6 months (subacute), >6 months (chronic).

Enrolled participants include stroke in acute, early subacute, subacute, and chronic phases. Most studies (62.53%) consider a homogeneous group of participants: 6.25% acute stroke participants [26,36,53], 16.70% early subacute stroke participants [18,21,33,34,35,43,52,55], 2.08% subacute stroke participants [64], and 37.50% chronic stroke participants [19,20,23,24,25,27,29,30,31,40,42,45,46,49,50,56,59,61]. The remaining studies (37.47%) include more than one group of stroke participants who differ in terms of time from the cerebral lesion to enrollment, e.g., early subacute and subacute. The distribution of studies across TSI classes is reported in Figure 2.

Most of the participants enrolled in the studies are inpatient (60.40%, [18,21,22,25,26,28,32,33,34,35,37,39,43,44,47,48,49,50,51,52,53,54,55,56,57,61,62,63,65]). Outpatient studies and those considering both inpatient and outpatient participation are 20.85% [19,20,23,24,27,29,42,58,59,64] and 6.25% [38,40,41], respectively. For the remaining 12.5%, there are no clear indications referring to the setting [30,31,36,45,46,60].

**Figure 3:**
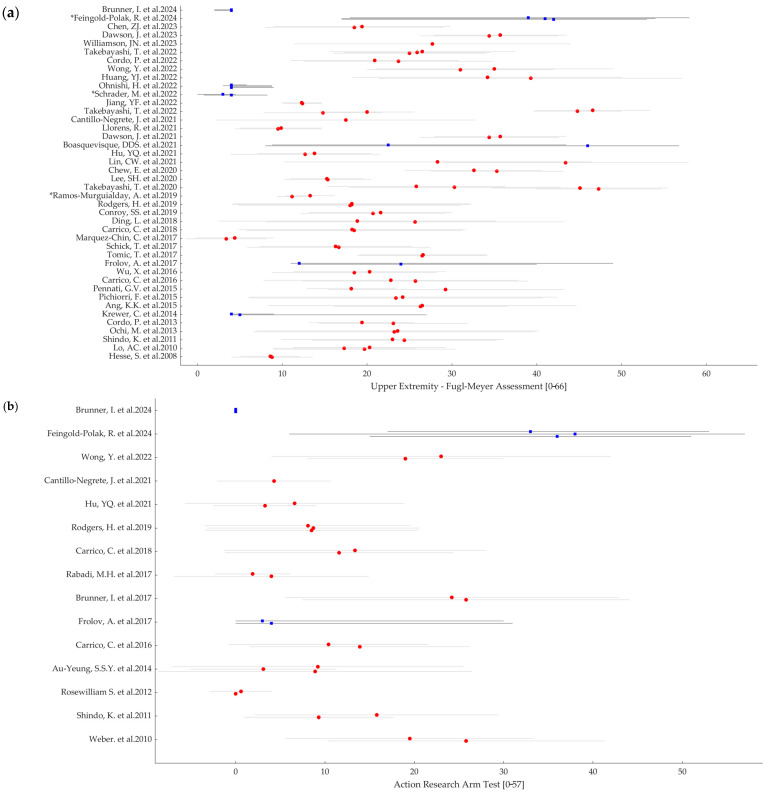
(**a**) Upper Extremity Fugl-Meyer Assessment (FMA) score: minimum score 0, maximum score 66 equal to normal. (**b**) Action Research Arm Test score: minimum score 0, maximum score 57 equal to normal. Red circle and grey line code for studies presenting FMA or ARAT score expressed as the mean (red circle) ± standard deviation (grey line). Blue square marker and black line code for studies presenting FMA or ARAT score expressed as the median (blue square) and first/third quartile (black line running from the first to the third quartile). (*) marker codes for two studies [54,64] that assess FMA out of a maximum of 60 score and codes for one study [42] that assesses FMA out of a maximum of 54 score.

Figure 3 summarizes the results about the severity of upper limb motor impairment upon enrollment based on FMA (panel a) and ARAT (panel b) scores at baseline. Forty-two studies assess baseline motor impairment in stroke participants by means of the FMA score, while in fifteen studies, the baseline assessment was performed by means of the ARAT score. As can be noted in Figure 3, both FMA and ARAT scores at baseline extend across the whole range, i.e., FMA: 21.71 ± 11.41 (mean ± SD across 42 studies) and ARAT: 12.28 ± 10.85 (mean ± SD across 15 studies). Of note, among the seven studies for which the mean or median FMA value falls below 10, three of them included acute patients [37,54,65].

The complete dataset about the characteristics of each participant enrolled in the study (e.g., individual FMA or ARAT scores) is available in 15% of studies.

**Figure 4:**
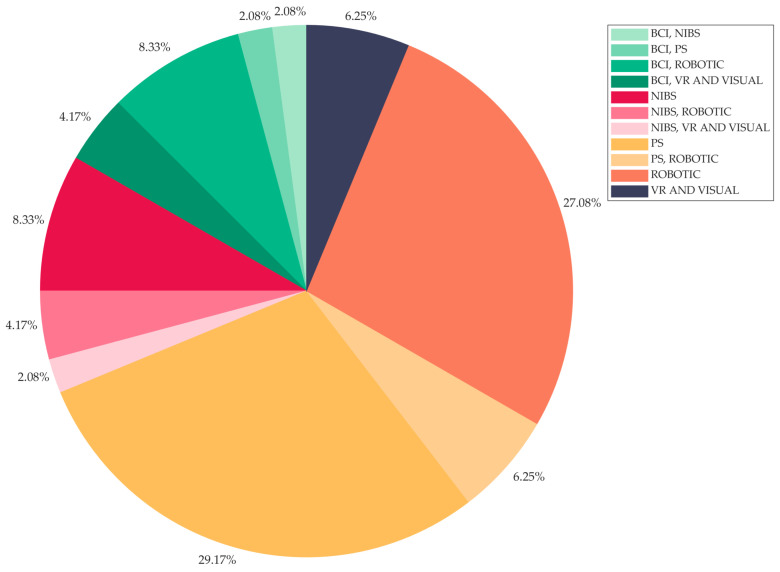
Distribution across studies (*n* = 48) of the technology-based rehabilitative intervention types, grouped as Brain–Computer Interface (BCI), Non-Invasive Brain Stimulation (NIBS), Peripheral Stimulation (PS), Robotic, Virtual Reality (VR) and Visual, and their combination.

The pie chart in Figure 4 shows the distributions of studies across technological rehabilitative intervention types. Rehabilitative interventions administered by means of robotic devices and peripheral stimulation and their combination cover more than half of the tested interventions: 27.08% [19,29,31,33,40,41,43,44,52,54,59,63,64], 29.17% [20,21,22,25,26,30,34,37,38,49,53,55,57,61], and 6.25% [18,24,58], respectively. Fewer studies focus on BCI-based and NIBS interventions, both combined with other intervention types, and Virtual Reality and Visual rehabilitative interventions: 16.66% (eight articles [27,28,32,42,45,47,51,65]), 14.58% (seven articles [23,36,48,50,56,60,62]), and 6.25% (three articles [35,39,46]), respectively.

The majority (52.10%) of rehabilitative intervention approaches require participant’s residual motor ability (active motor exercise by the participants) [19,20,21,24,27,29,30,31,33,35,37,39,40,42,46,49,50,55,57,58,59,61,62,63,64]; 10.40% are categorized as “Yes whenever possible”, referring to conditions foreseeing active motor exercise when feasible with the technology providing assistance as needed (e.g., robotics) [18,41,43,44,52]; and 37.50% do not require any active motor action from the participants [22,23,25,26,28,32,34,36,38,45,47,48,51,53,54,56,60,65].

Regarding the dose of rehabilitative intervention, the studies differ in terms of both minutes of each training session, 40 min (median) and 30–60 min (Q1–Q3), and number of training sessions (21.78 ± 14.39, mean ± SD), ranging from 12 sessions (Q1) to 28 sessions (Q3). The overall dose of intervention, resulting from the multiplication of minutes per session and number of sessions, is 13h (median) and 8–27 h (Q1–Q3).

Table 2 shows the number of studies in which each surveyed primary and secondary outcome is used either as primary or secondary. As for the primary outcome, the Upper Extremity FMA results are the most frequent scale administered to assess the efficacy of the rehabilitative interventions (38 on 48 studies, 79.17%) [18,19,21,23,24,25,27,28,29,30,31,32,33,34,37,39,40,42,43,44,45,46,47,49,50,51,52,53,54,55,56,58,59,60,61,62,63,64]. Conversely, several evaluations, such as clinical/functional as well as instrumental, are considered for the secondary outcomes. Among the clinical/functional secondary outcomes, the most frequent are the Stroke Impact Scale [66] (22.92% of studies, [19,24,31,38,40,41,44,48,49,58,59]), the Barthel Index [67] (22.92% of studies, [22,25,33,34,39,41,47,48,53,57,63]), the Wolf Motor Function Test [68] (20.83% of studies, [19,30,31,33,40,44,49,50,56,61]), and the Action Research Arm Test [14] (16.67% of studies, [20,21,26,30,38,47,51,59]); transcranial magnetic stimulation, electroencephalographic, electromyographic, kinematic, and kinetic parameters are considered as brain and motor outcomes in 27.08% of the studies [22,28,42,45,47,51,53,58,59,60,62,63,64].

In 37.50% of the studies, the participants are followed-up from 2 weeks to 9 months after the end of the rehabilitation, i.e., 3 months (median) and 3–5 months (Q1–Q3), [18,19,22,25,26,27,30,31,35,36,38,41,44,45,48,49,61]. No follow-up evaluations are reported in 62.50% of the studies.

As for the comparison conditions, most studies (81.25%) are two-arm RCTs. The remaining 18.75% of studies [19,26,41,43,52,55,59,62,64] compare more than two groups, up to four groups in [55]. For the two-arm studies, the control conditions that are most commonly observed can be categorized as follows:

- Sham Stimulation/Control (applies in NIBS/PS and BCI studies, referring to conditions where the participants are induced to believe they are receiving stimulation or controlling a BCI system while they are not): 28.20% of studies [25,30,32,36,38,42,45,48,49,56,61];
- Similar intervention “without technology” (e.g., mirror therapy in contrast to VR-based mirror therapy): 17.95% of studies [20,21,28,46,47,54,57];
- Usual care: 17.95% of studies [22,31,37,50,53,63,65];
- Dose equivalent upper limb training (dose equivalent therapy session focused on the upper limb, considered an add-on to usual care): 10.26% of studies [33,35,39,43];
- Different combinations of technology-based approaches: 10.26% of studies [27,29,34,40];
- Different technology: 7.69% of studies [18,24,44];
- Different parameters of the same technology (e.g., different robotic assistance, anodal vs. cathodal transcranial direct-current stimulation): 7.69% of studies [23,52,58].

For studies comparing more than two groups, different interventions are mostly compared to the usual care control condition. Further details about the comparator employed in each study can be found in the Supplementary Materials (Table S2).

**Figure 5:**
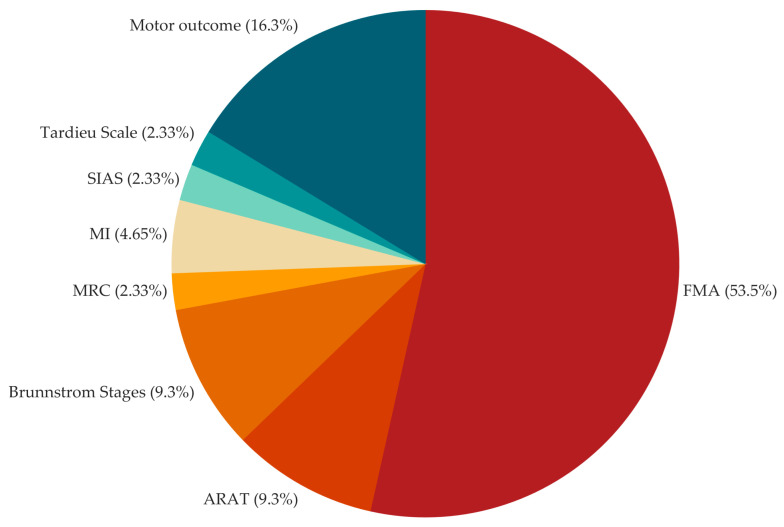
Distribution across studies (n = 43) of the parameters (clinical/functional scale or motor-related evaluation) used for the enrollment inclusion criteria definition and grouped as UE-FMA: Upper Extremity Fugl-Meyer Assessment; ARAT: Action Research Arm Test; MRC: Medical Research Council Scale; MI: Motricity Index; and SIAS: Stroke Impairment Assessment Set; and Motor outcome, which concerns range of motion evaluation and evaluation of ability/inability to perform a specific task.

**Figure 6:**
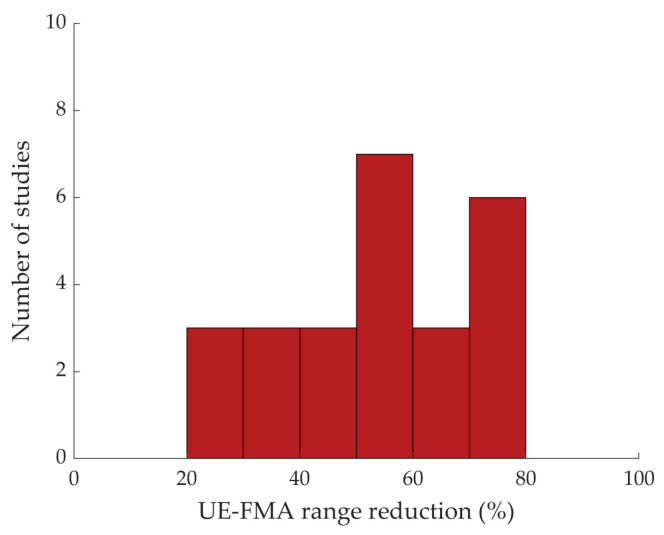
Histogram of number of studies that define inclusion criteria by means of Upper Extremity Fugl-Meyer Assessment (UE-FMA) score, reported as a function of the UE-FMA range reduction achieved by defining inclusion criteria for participant enrollment.

As shown in Table 1, most studies (89.58%) employ inclusion criteria related to upper limb impairment for participant enrollment [18,19,20,21,22,23,24,25,26,27,30,31,33,34,35,36,37,38,40,41,42,43,44,45,46,47,49,50,51,52,53,54,55,56,57,58,59,60,61,62,63,64,65]. Figure 5 shows the inclusion criteria and their frequency across studies (43 studies include inclusion criteria). More than half of the studies define the inclusion criteria by means of an FMA score (53.50% of studies, [18,19,23,27,31,33,34,37,38,40,44,45,46,49,50,54,58,59,60,61,62,63,64]) or ARAT (9.30% of studies, [22,35,41,65]). Nevertheless, evaluations based on motor outcome, i.e., range of motion or ability/inability to perform a specific task, such as that involved in the study protocol, are taken into account in 16.3% of studies [20,21,24,30,42,47,57] as well as spasticity-related scales, i.e., Brunnstrom stages and Tardieu scale, in 11.63% of studies [25,43,52,53,56]. Specifically, for the FMA inclusion criteria, Figure 6 shows the reduction gained by defining the inclusion criteria for participant enrollment. That reduction, expressed as percentage, is computed as the one’s complement of the ratio between the FMA range defined as in the inclusion criteria and the whole FMA range (0–66), according to the following formula:

$$FMA\;range\;reduction=100\times (1-\frac{FMA\;range\;defined\;as\;inclusion\;criteria}{66})$$

In seven studies, we observed a reduction between 60% and 80% [18,33,34,37,44,50,54]; among them is the study by Schrader et al. [54] in which inclusion criteria have been defined on the basis of the hand section of the FMA scale (maximum value 14). Most of the studies only achieve a 50% reduction [19,31,40,49,60,61,63], thus including in the same analyses participants who differ in FMA score up to 33 points (50% of the whole FMA range [0 66]), for example, in the range [8 38] in the study by Chen and colleagues in [63].

As for the severity of the upper limb impairment at baseline for the stratification of participants in secondary analyses, 25.00% of studies exploit such an evaluation [24,31,34,35,39,40,41,43,45,52,61,63], defining two or more levels of the variable: FMA (nine studies out of twelve) [24,31,34,39,40,43,52,61,63] and ARAT [41], motor evoked potentials, [45] and range of motion [35] (one per study).

As for the key findings of the trials, (i) 41.67% report positive results, i.e., the studies confirm the hypothesis of efficacy of a given intervention via between-group analysis [21,26,28,30,31,33,37,38,39,46,49,50,53,54,55,58,60,61,62,63]; and (ii) 31.25% report positive results on secondary analyses, i.e., between-group statistical analyses evaluated for sub-items of the primary/secondary outcomes or considering sub-groups of the population under investigation confirm the original hypothesis or if within-group statistical analyses evaluated for the primary/secondary outcomes reveal a statistical improvement only for the experimental group [19,23,24,25,32,34,40,42,43,45,47,52,56,59,64]. Negative results are reported by 25.00% of studies, i.e., the target intervention is not superior to the control condition [18,20,22,27,29,35,36,41,48,51,57,65].

#### 3.3.2. Association Results

**Figure 7:**
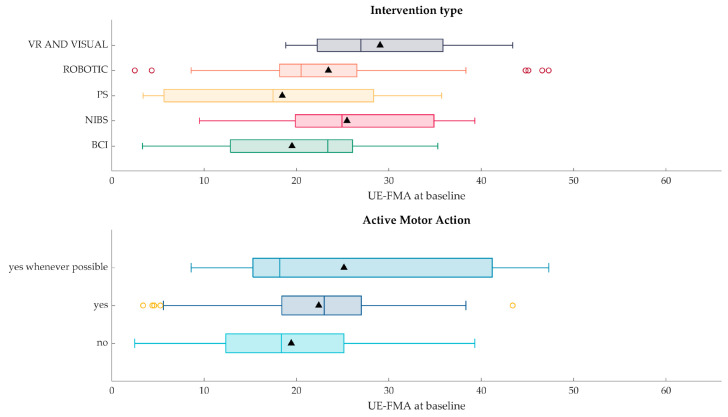
Distribution across studies (n = 42) of UE-FMA at baseline, grouped by intervention type (**top**) and motor action required from the participant (**bottom**). The triangle-up marker codes for the average of each distribution and the circle codes for outliers. Intervention types were categorized as BCI (Brain–Computer Interface), NIBS (Non-Invasive Brain Stimulation), PS (peripheral stimulation), Robotic, and VR (Virtual Reality) and Visual. Each study is assigned to a single category. Studies that investigate combinations of technology-based interventions, e.g., BCI and PS or BCI and Robotic, have been assigned to the most significant intervention type according to the study design, e.g., BCI and PS have been assigned to the BCI category when PS was employed in the control condition as well, thus BCI control resulted to be the core of the rehabilitative intervention in the study. Active motor actions are categorized as no/yes/yes whenever possible.

Figure 7 shows, for each type of intervention (top panel in the figure) and motor action required from the participant by the intervention itself (bottom panel in the figure), the upper limb impairment severity (UE-FMA) of the participants recruited in those studies. Each study has been categorized both according to the intervention type (analysis presented in the top panel) and motor action required (analysis presented in the bottom panel). The data from studies belonging to the same category, e.g., intervention type PS, are pooled together and summarized by means of box charts.

As for the intervention type, most studies seem to cover almost half of the UE-FMA scale scores. On average, PS-based interventions concern participants with a slightly lower UE-FMA value (18.46 ± 11.55) than the other interventions (BCI: 19.52 ± 9.72, NIBS: 25.47 ± 10.67, Robotic: 23.46 ± 10.74, VR and Visual: 29.05 ± 10.36). Robotic interventions, as shown from the distribution outliers, include studies with UE-FMA values both lower than 5 and higher than 40. As for motor action required from the participant, studies in which the experimental protocol does not require action of the participant enroll participants with UE-FMA on average lower (19.44 ± 10.62) than studies either requiring active motor action (22.41 ± 9.94) or whenever possible (25.14 ± 14.09). Of note, studies that require active motor actions enroll participants with reduced motor action ability (FMA < 5) as well as participants with moderate motor impairment (FMA = 43).

## 4. Discussion

In this scoping review, we provide a portrait of the current evidence derived from RCTs investigating the efficacy of technology-based interventions targeting upper limb motor recovery in patients with severe impairment after stroke. Our main aim is to verify the actual severity of the included patients enrolled in such trials to confirm or refute the anecdotal notion of extreme variability in baseline motor impairment, which might be responsible for the lack of solid evidence supporting the efficacy of such interventions [9].

We included papers reporting FMA or ARAT or both to assess upper limb motor impairment (Table S1, Supplementary Materials) since they are commonly employed to investigate the efficacy of the rehabilitative treatment [69]. Our results (Table 2) show that FMA is by far the most commonly employed as the primary outcome measure, followed by ARAT; other measures that are specific for upper limb function, such as BBT, WMFT, and MAL, are more commonly employed as the secondary outcome.

According to our descriptive analyses on FMA and ARAT values upon enrollment, we verified that the included patients altogether virtually cover the whole range of both scales (Figure 3a,b). That is, these RCTs that were originally implemented to investigate the efficacy of rehabilitative interventions designed for severe patients often include patients with mild to moderate deficits as well. This occurs despite the fact that almost 90% of the studies actually defined the inclusion criteria based on severity. Actually, there is no unique definition of severity, even for the same assessment scale. To reach such a univocal definition of motor severity is beyond the scope of our work; however, our review allows for us at least to provide a picture of the current state of the art in this aspect. Among those studies in which the inclusion criteria were based on severity, approximately 50% employed FMA for such a definition. However, the references for the proposed stratification did not converge on a unique subdivision. For example, several papers refer to Fugl-Meyer et al. [13] or Gladstone et al. [70] to justify the use of cut-off values for their inclusion criteria or stratification analyses, but no subdivision is provided in either of these papers. Woodbury et al. [71] suggest a cut-off below 19 for severe patients and of 47 for moderately impaired patients, which is applied as an inclusion criterion by Carrico et al. [38]. Ding et al. [39] apply the clustering suggested in Woytowicz et al. [72] to define severity in patients with an FMA < 35. Conroy et al. [40] apply a cut-off of 25 as suggested by Luft et al. [73]. As a possible explanation for such a wide range of severity among the enrolled patients, we hypothesized that the technology in the study could play a role. For example, some robotic devices cannot be proposed to patients with severe spasticity, or an electromyographically triggered orthosis cannot be activated if patients have no residual movement in the target muscles. Thus, we categorized the papers according to the proposed technology. Electrical or magnetic stimulation of peripheral structures (PS) is the most represented technology in our review, followed by robotics. These two or their combinations represent approximately 63% of the studies. The BCI and NIBS (often in combination with other devices) are the topics of approximately 31% of the studies, while the less represented technology is VR, with just approximately 6% of the papers. To further characterize the interventions according to the fact that they required (or not) active motor actions from the patients, we found out that more than 50% of the interventions required some residual motor ability from the patients. This could be an explanation for the trend to include “not-so-severe” patients in order to increase trial feasibility. Nevertheless, we did not observe a clear difference in the severity of the included patients when we divided the studies according to the technology or to the presence/absence of an active motor exercise. The distributions of patients’ severity (Figure 7) qualitatively show a tendency towards the more severe range for the interventions based on PS and for interventions not requiring active motor tasks. We also highlighted the heterogeneity of included patients in terms of time since injury. While the majority of studies targeted chronic patients exclusively (37.5%), the studies including mixed groups altogether reached a similar percentage (37.47%). It is well known that brain plasticity that underlies motor recovery is time dependent, and the recovery potential is different according to the post-stroke phase [74]. Moreover, changes occurring in peripheral structures along recovery (e.g., spasticity, complications related to reduced mobility) have a direct impact on the motor status (and, thus, potential motor outcome), further increasing the complexity of the overall picture.

Regarding the study design, we also pointed out extreme variability in terms of the dose/intensity of treatment, type of comparator, and sample size. In almost 20% of the two-arm studies, “usual care” is the only control condition, while an active comparator would be most desirable. Different dosages and very different sample sizes (ranging from 10 to almost 800 participants) limit the potential impact of our attempt to interpret the results of this scoping review that takes into account such a wide scenario of clinical studies. An indirect result of our scoping review derives from the papers that we excluded for reporting study protocols (n = 13). These papers were all published between 2017 and 2023 [75,76,77,78,79,80,81,82,83,84,85,86,87], with ten of them being subsequent to 2020, testifying to a progressive increase in the efforts dedicated to rigorous clinical trial design in this field of translational research.

The ultimate goal of our reviewing work is to improve the design of RCTs to boost the translation of rehabilitative technologies into clinical practice. Indeed, the majority of the selected studies report positive results, indicating the efficacy of the proposed technology. However, approximately 30% of the studies required secondary analyses to support the benefit derived from the intervention in the study. Some of them report positive results on secondary outcomes, e.g., SIS and WMFT [19,40], and/or outcome sub-items, e.g., ARAT, FMA, and MAS items [23,43]. Some required subgroup analyses, e.g., proving efficacy only in severe patients [34,43,45,52]. The remaining 25% of openly negative studies (showing non-superiority of the proposed intervention) is likely underestimated, as it is known that publishing a negative result is harder and often occurs only in the case of very well designed and well conducted, large clinical trials.

A further observation derived from our work is that, along with the established clinical and functional scales employed as primary or secondary outcomes (Table 2), 16 papers applied objective measurements of brain and motor activity as a means to verify the effects of the intervention on motor system performance. Advanced analyses on electroencephalographic [28,51] and transcranial magnetic stimulation recordings [45,51,60,62], electromyographic, kinematic, and kinetic data [22,26,42,47,51,53,58,59,62,63,64] were performed in these studies to identify modifications in motor system performance subserving a favorable motor outcome derived from the intervention in the study. This suggests that technology is not only employed for the design of rehabilitative interventions but may play a crucial role in improving the outcome assessment with more objective, measurable, and reproducible parameters that may serve as biomarkers of motor recovery.

This scoping review has several limitations, mostly related to the heterogeneity of the included studies in terms of numerosity, characteristics of the enrolled patients, trial design, implementation, and reporting. We considered papers published between 2008 and 2024, observing a progressive improvement in all of these aspects, which will hopefully be fruitful in the upcoming years to properly address some of the issues that we raised here and that remain yet unanswered. Only seven studies [28,29,30,45,51,56,60] provide a complete dataset documenting the individual severity of the enrolled patients, which would allow for a statistical analysis on how effective the interventions were according to a homogeneous stratification of patients based on severity. The policies for data availability are also progressively pointing towards safe data sharing according to internationally agreed upon General Data Protection Regulations.

## 5. Conclusions

We represented the current evidence derived from RCTs investigating the efficacy of technology-based interventions targeting upper limb motor recovery in patients with severe impairment after stroke. Our aim was to shed light on the problems of the current research in rehabilitation technologies to ultimately boost the translational success of such approaches. There is undoubtedly a need for patient stratification upon enrollment to selectively direct resources to the patients who will benefit the most from a given approach. The correct taxonomy of patient severity and the related correct reporting in clinical trials could significantly improve the transnationality and contextualization of the results obtained, avoiding biases that could affect potential effectiveness. Only severe patients should be recruited for the clinical validation of devices that are designed specifically for them, while the design and development of technologies with adequate sensorimotor and cognitive stimulation would probably increase their salience (and effectiveness) for less affected subjects. Upon the improvement in the design, implementation, and reporting of clinical trials, subsequent systematic reviews will probably help in identifying strong evidence and, thus, evidence-based indications for clinicians operating in the field of neurorehabilitation.

## Figures and Tables

**Table 1:** Studies included in this review. The following data are reported: name of first author and publication year, population sample size (number of participants per group, whenever available), severity of the impairment assessed via UE-FMA and ARAT (per group, whenever available), if inclusion criteria related to the upper limb impairment for the participant enrolment (IC) were defined, time since injury, intervention type, active motor action required, comparator, primary outcome measures, and key findings. UE-FMA and ARAT values are reported as the mean ± standard deviation or median and interquartile range (Q1–Q3).

First Author Name, Year of Publication	Population Sample Size (Participants per Group)	Severity of the Impairment (UE-FMA and/or ARAT per Group)	IC	Time Since Injury	Intervention Type	Active Motor Action Required	Comparator	Primary Outcome Measures	Key Findings
Hesse, 2008 [18]	EG: 27 CG: 27	UE-FMA	Yes	ES	PS, Robotic	Yes, whenever possible	Different Technology	UE- FMA	Neg
		EG: 8.8 ± 4.5							
		CG: 8.6 ± 3.5							
Lo, 2010 [19]	EG: 47 CG1: 46 CG2: 27	UE-FMA	Yes	C	Robotic	Yes	Dose Equivalent UL Training, Usual Care	UE- FMA	Pos On Sec
		EG: 19.7 ± 10.7							
		CG1: 17.3 ± 8.4							
		CG2: 20.3 ± 9.0							
Weber, 2010 [20]	EG: 10 CG: 13	ARAT	Yes	C	PS	Yes	Without Technology	MAL	Neg
		EG: 19.5 ± 13.9							
		CG: 25.8 ± 15.5							
Shindo, 2011 [21]	EG: 10 CG: 10	UE-FMA	Yes	ES	PS	Yes	Without technology	UE-FMA	Pos
		EG: 24.4 ± 10.9							
		CG: 23.0 ± 13.1							
		ARAT							
		EG: 9.3 ± 8.4							
		CG: 15.8 ± 13.7							
Rosewilliam, 2012 [22]	EG: 31 CG: 36	ARAT	Yes	A/ES	PS	No	Usual Care	ARAT	Neg
		EG: 0.0 ± 0.0							
		CG: 0.6 ± 3.5							
Ochi, 2013 [23]	EG: 9 CG: 9	UE-FMA	Yes	C	NIBS, Robotic	No	Different Parameters	UE-FMA	Pos On Sec
		EG: 23.2 ± 16.6							
		CG: 23.6 ± 16.7							
Cordo, 2013 [24]	EG1: 22 EG2: 21	UE-FMA	Yes	C	PS, Robotic	Yes	Different Technology	UE-FMA	Pos On Sec
		EG1: 23.1 ± 8.8							
		EG2: 19.4 ± 6.2							
Krewer, 2014 [25]	EG: 31 CG: 32	UE-FMA	Yes	C	PS	No	Sham Stimulation	MTS UE-FMA	Pos On Sec
		EG: 5 (4–27)							
		CG: 4 (4–9)							
Au-Yeung, 2014 [26]	EG: 29 CG1: 21 CG2: 23	ARAT	Yes	A	PS	No	Sham Stimulation, Usual Care	Force measures	Pos
		EG: 8.9 ± 17.6							
		CG1: 3.1 ± 8.2							
		CG2: 9.2 ± 16.3							
Ang, 2015 [27]	EG: 11 CG: 14	UE-FMA	Yes	C	BCI, Robotic	Yes	Different Combination	UE-FMA	Neg
		EG: 26.3 ± 10.3							
		CG: 26.5 ± 18.2							
Pichiorri, 2015 [28]	EG: 14 CG: 14	UE-FMA	No	ES/S	BCI, VR and Visual	No	Without Technology	UE-FMA	Pos
		EG: 23.4 ± 17.3							
		CG: 24.2 ± 18.2							
Pennati, 2015 [29]	EG1: 8 EG2: 7	UE-FMA	No	C	Robotic	Yes	Different Combination	UE-FMA BBT FIM MAS	Neg
		EG1: 29.25 ± 13.91							
		EG2: 18.14 ± 5.27							
Carrico, 2016 [30]	EG: 18 CG: 18	UE-FMA	Yes	C	PS	Yes	Sham Stimulation	UE-FMA	Pos
		EG: 25.7 ± 13.3							
		CG: 22.8 ± 15.0							
		ARAT							
		EG: 13.9 ± 12.3							
		CG: 10.4 ± 11.2							
Wu, 2016 [31]	EG: 99 CG: 28	UE-FMA	Yes	C	Robotic	Yes	Usual Care	UE-FMA	Pos
		EG: 18.5 ± 9.7							
		CG: 20.3 ± 9.0							
Frolov, 2017 [32]	EG: 55 CG: 19	UE-FMA	No	S/C	BCI, Robotic	No	Sham Control	UE-FMA ARAT	Pos On Sec
		EG: 24.0 (12.0–40.0)							
		CG: 12.0 (11.0–49.0)							
		ARAT							
		EG: 4.0 (0.0–31.0)							
		CG: 3.0 (0.0–30.0)							
Tomic, 2017 [33]	EG: 13 CG: 13	UE-FMA	Yes	ES	Robotic	Yes	Dose Equivalent UL Training	UE-FMA	Pos
		EG: 26.5 ± 7.7							
		CG: 26.6 ± 7.5							
Schick, 2017 [34]	EG: 16 CG: 17	UE-FMA	Yes	ES	PS	No	Different Combination	UE-FMA	Pos On Sec
		EG: 16.67 ± 10.80							
		CG: 16.29 ± 9.00							
Brunner, 2017 [35]	EG: 57 CG: 55	ARAT	Yes	ES	VR and Visual	Yes	Dose Equivalent UL Training	ARAT	Neg
		EG: 25.8 ± 18.3							
		CG: 24.2 ± 18.6							
Rabadi, 2017 [36]	EG: 8 CG: 8	ARAT	Yes	A	NIBS	No	Sham Stimulation	ARAT	Neg
		EG: 4.0 ± 10.9							
		CG: 1.9 ± 4.2							
Marquez-Chin, 2017 [37]	EG: 10 CG: 11C	UE-FMA	Yes	A/ES	PS	Yes	Usual Care	FIM UE-FMA	Pos
		EG: 3.4 ± 4.8							
		CG: 4.4 ± 4.6							
Carrico, 2018 [38]	EG: 33 CG: 22	UE-FMA	Yes	S/C	PS	No	Sham Stimulation	WMFT	Pos
		EG: 18.48 ± 12.75							
		CG: 18.23 ± 13.34							
		ARAT							
		EG: 11.58 ± 12.80							
		CG: 13.36 ± 14.68							
Ding, 2018 [39]	EG: 38 CG: 41	UE-FMA	No	S/C	VR and Visual	Yes	Dose Equivalent UL Training	UE-FMA	Pos
		EG: 25.66 ± 17.63							
		CG: 18.85 ± 16.38							
Conroy, 2019 [40]	EG: 22 CG: 19	UE-FMA	Yes	C	Robotic	Yes	Different Combination	UE-FMA	Pos On Sec
		EG: 20.7 ± 8.5							
		CG: 21.6 ± 8.5							
Rodgers, 2019 [41]	EG: 239 CG1: 246 CG2: 223	UE-FMA	Yes	S/C	Robotic	Yes, whenever possible	Dose Equivalent UL Training, Usual care	ARAT	Neg
		EG: 18.0 ± 13.1							
		CG1: 18.2 ± 14.1							
		CG2: 18.2 ± 13.9							
		ARAT							
		EG: 8.5 ± 11.9							
		CG1: 8.7 ± 11.9							
		CG2: 8.1 ± 11.5							
Ramos-Murguialday, 2019 [42]	EG: 16 CG: 12	UE-FMA	Yes	C	BCI, Robotic	Yes	Sham Control	UE-FMA (54)	Pos On Sec
		EG: 11.16 ± 1.73							
		CG: 13.29 ± 2.86							
Takebayashi, 2020 [43]	EG: 30 CG: 26	UE-FMA	Yes	ES	Robotic	Yes, whenever possible	Dose Equivalent UL Training	UE-FMA	Pos On Sec
		EG: 47.3 ± 7.4 (mild)							
		CG: 45.1 ± 19.4 (mild)							
		EG: 30.3 ± 12.5 (moderate)							
		CG: 25.8 ± 10.5 (moderate)							
		EG: 16.1 ± 10.5 (severe)							
		CG: 14.8 ± 4.7 (severe)							
Lee, 2020 [44]	EG1: 19 EG2: 19	UE-FMA	Yes	S/C	Robotic	Yes, whenever possible	Different Technology	UE-FMA WMFT	(*)
		EG1: 15.37 ± 5.14							
		EG2: 15.26 ± 4.37							
Chew, 2020 [45]	EG: 10 CG: 9	UE-FMA	Yes	C	BCI, NIBS	No	Sham Stimulation	UE-FMA	Pos On Sec
		EG: 35.3 ± 7.8							
		CG: 32.6 ± 8.1							
Lin, 2021 [46]	EG: 9 CG: 9	UE-FMA	Yes	C	VR and Visual	Yes	Without technology	UE-FMA	Pos
		EG: 43.4 ± 14.5							
		CG: 28.3 ± 18.1							
Hu, 2021 [47]	EG: 7 CG: 5	UE-FMA	Yes	S/C	BCI, VR and Visual	No	Without technology	UE-FMA	Pos On Sec
		EG: 12.70 ± 8.80							
		CG: 13.80 ± 6.65							
		ARAT							
		EG: 3.29 ± 5.79							
		CG: 6.60 ± 12.29							
Boasquevisque, 2021 [48]	EG: 15 CG: 15	UE-FMA	No	A/ES	NIBS	No	Sham Stimulation	Safety (**)	Neg
		EG: 46 (8–56.8)							
		CG: 22.5 (8.8–43.5)							
Dawson, 2021 [49]	EG: 53 CG: 54	UE-FMA	Yes	C	PS	Yes	Sham Stimulation	UE-FMA	Pos
		EG: 34.4 ± 8.2							
		CG: 35.7 ± 7.8							
Llorens, 2021 [50]	EG: 14 CG: 15	UE-FMA	Yes	C	NIBS, VR and Visual	Yes	Usual Care	UE-FMA	Pos
		EG: 9.50 ± 5.11							
		CG: 9.87 ± 4.82							
Cantillo-Negrete, 2021 [51]	10 crossover study	UE-FMA	Yes	S/C	BCI, Robotic	No	Usual Care	UE-FMA	Neg
		17.5 ± 15.3							
		ARAT							
		4.3 ± 6.4							
Takebayashi, 2022 [52]	EG1: 17 EG2: 13	UE-FMA	Yes	ES	Robotic	Yes, whenever possible	Different Parameters	UE-FMA WMFT	Pos On Sec
		EG1: 14.8 ± 7.0 (severe)							
		EG2: 20.0 ± 5.6 (severe)							
		EG1: 44.8 ± 5.2 (moderate)							
		EG2: 46.6 ± 6.8 (moderate)							
Jiang, 2022 [53]	EG: 24 CG: 20	UE-FMA	Yes	A	PS	No	Usual Care	UE-FMA	Pos
		EG: 12.38 ± 2.26							
		CG: 12.30 ± 2.39							
Schrader, 2022 [54]	EG: 14 CG: 10	UE-FMA	Yes	A/S/C	Robotic	No	Without Technology	UE-FMA (60)	Pos
		EG: 4.00 (0.75–8.25)							
		CG: 3.00 (0.00–4.50)							
Ohnishi, 2022 [55]	EG1: 25 EG2: 22 EG3: 26 CG: 26	UE-FMA	Yes	ES	PS	Yes	Different Parameters, Usual Care	SIAS UE-FMA MAS FIM	Pos
		EG1: 4.0 (4.0–9.0)							
		EG2: 4.0 (4.0–8.8)							
		EG3: 4.0 (3.0–8.8)							
		CG: 4.0 (4.0–5.8)							
Huang, 2022 [56]	EG: 13 CG: 11	UE-FMA	Yes	C	NIBS	No	Sham Stimulation	UE-FMA	Pos On Sec
		EG: 39.3 ± 17.9							
		CG: 34.2 ± 15.9							
Wong, 2022 [57]	EG: 15 CG: 15	UE-FMA	Yes	A/ES	PS	Yes	Without Technology	ARAT	Neg
		EG: 31 ± 11							
		CG: 35 ± 14							
		ARAT							
		EG: 19 ± 11							
		CG: 23 ± 19							
Cordo, 2022 [58]	EG: 44 CG: 39	UE-FMA	Yes	ES/S	PS, Robotic	Yes	Different Parameters	UE-FMA	Pos
		EG: 20.9 ± 9.9							
		CG: 23.7 ± 11.2							
Takebayashi, 2022 [59]	EG1: 42 EG2: 39 CG: 36	UE-FMA	Yes	C	Robotic	Yes	Without Technology	UE-FMA	Pos On Sec
		EG1: 25.9 ± 8.6							
		EG2: 26.5 ± 11.0							
		CG: 25.0 ± 0.9							
Williamson, 2023 [60]	8 crossover study	UE-FMA	Yes	ES/S/C	NIBS	No	Different Parameters, Sham Stimulation	UE-FMA	Pos
		27.7 ± 16.3							
Dawson, 2023 [61]	EG: 53 CG: 55	UE-FMA	Yes	C	PS	Yes	Sham Stimulation	UE-FMA	Pos
		EG: 34.4 ± 8.2							
		CG: 35.7 ± 7.8							
Wang, 2023 [62]	EG1: 23 EG2: 23 CG: 23	UE-FMA	Yes	ES/S	NIBS, Robotic	Yes	Different Technology, Usual care	UE-FMA BI	Pos
		EG1: 9 (IQR: 12)							
		EG2: 11 (IQR: 8)							
		CG: 14 (IQR: 16)							
Chen, 2023 [63]	EG: 40 CG: 40	UE-FMA	Yes	A/ES	Robotic	Yes	Usual Care	UE-FMA	Pos
		EG: 18.5 ± 10.5							
		CG: 19.4 ± 10.4							
Feingold-Polak, 2024 [64]	EG1: 10 EG2: 8 CG: 8	UE-FMA (60)	Yes	S	Robotic	Yes	Different Technology, Usual Care	UE-FMA (60) ARAT MAL SIS	Pos On Sec
		EG1: 42 (17–53)							
		EG2: 41 (17–54)							
		CG: 39 (18–58)							
		ARAT							
		EG1: 36 (15–51)							
		EG2: 38 (6–57)							
		CG: 33(17–53)							
Brunner, 2024 [65]	EG: 15 CG: 20	UE-FMA	Yes	A/ES	BCI, PS	No	Usual Care	ARAT	Neg
		EG: 4 (2–4)							
		CG: 4 (2–4)							
		ARAT							
		EG: 0 (0–0)							
		CG: 0 (0–0)							

Legend: A: Acute phase (≤1 month); ARAT: Action Research Arm Test; BCI: Brain–Computer Interface; BBT: Box and Block Test; BI: Barthel Index; CG: Control Group; C: Chronic phase (>6 months); EG: Experimental Group; ES: Early Subacute phase (≤3 months); FIM: Functional Independence Measure; IQR: inter-quartile range; MAL: Motor Activity Log; MAS: Modified Ashworth Scale; MTS: Modified Tardieu Scale; Neg: Negative key findings; NIBS: Non-Invasive Brain Stimulation; Pos: Positive key findings; Pos On Sec: Positive findings on secondary analyses; PS: Peripheral Stimulation; S: Subacute phase (≤6 months); SIAS: Stroke Impairment Assessment Set; SIS: Stroke Impact Scale; UL: upper limb; UE-FMA: Upper extremity Fugl-Meyer Assessment; VR: Virtual Reality; WMFT: Wolf Motor Function Test. (*) Lee et al., 2020 [44]: we did not define key findings because the hypothesis about the effectiveness of the intervention EG1 compared to the EG2 is not clear. (**) Boasquevisque et al., 2021 [48]: safety is defined as primary outcome, but authors stated the primary outcome results were published elsewhere. (60) or (54): the expression (60) and (54) codes for UE-FMA assessed on 60 or 54, respectively.

**Table 2:** List of the primary and secondary outcomes and number of studies that consider each one as a primary (column on the left side) or secondary (column on the right side) outcome.

Outcome	As Primary Number of Studies	As Secondary Number of Studies
Upper Extremity Fugl-Meyer Assessment	38	5
Action Research Arm Test	8	8
Wolf Motor Function Test	3	10
Functional Independence Measure	3	2
Modified Ashworth Scale	2	9
Motor Activity Log	2	6
Stroke Impact Scale	1	11
Barthel Index	1	11
Box and Block Test	1	4
Stroke Impairment Assessment Set: knee–mouth and finger function test	1	
Modified Tardieu Scale	1	
Goal Attainment Scaling		2
Motricity Index		2
Medical Research Council Scale		2
National Institutes of Health Stroke Scale		2
Finger–Nose Test		1
Jebsen–Taylor Hand Function Test		1
Hamilton Depression Scale		1
Mental Rotation Task		1
Modified Rankin Scale		1
Montreal Cognitive Assessment		1
Nine-hole peg test		1
Nottingham Sensory Assessment		1
Numeric Rating Scale Pain		1
Rancho Los Amigos Scale		1
Rivermead Assessment of Somatosensory Performance		1
Stroke Specific Quality of Life Scale		1
Motor Outcome (kinematic, kinetic, electromyographic parameters)	1	10
Brain Outcome (transcranial magnetic stimulation and electroencephalographic parameters)		5
Safety (adverse events)		1

## Data Availability

No new data were created or analyzed during this study. Data sharing is not applicable to this article.

## Supplementary Materials

The following supporting information can be downloaded at: https://www.mdpi.com/article/10.3390/jcm13185414/s1, Table S1: Database Search Strategies. Table S2: Experimental and control intervention details of the studies included in the review.
