# Supplementary material for: A Scoping Review of Technology-Based Approaches for Upper Limb Motor Rehabilitation after Stroke: Are We Really Targeting Severe Impairment?
Source: J Clin Med. 2024 Sep 12;13(18):5414. doi: 10.3390/jcm13185414 (PMC11432574; doi:10.3390/jcm13185414)
Supplement: Supplementary file 1 [file jcm-13-05414-s001.zip › jcm-3179295-supplementary.pdf]

Table S1 - Database Search Strategies

Database	Search Strategy		Results
Scopus	#1	TITLE-ABS-KEY(stroke) AND TITLE-ABS-KEY(severe) AND TITLE-ABS-KEY(rehabilitation) AND (TITLE-ABS-KEY("Action Research Arm Test") OR TITLE-ABS-KEY(ARAT) OR TITLE-ABS-KEY("Fugl-Meyer Assessment") OR TITLE-ABS-KEY(FMA)) AND (TITLE-ABS-KEY("randomized controlled trial") OR TITLE-ABS-KEY("randomized clinical trial")) AND (TITLE-ABS-KEY("upper limb") OR TITLE-ABS-KEY(hand))	91
	#2	TITLE-ABS-KEY(stroke) AND TITLE-ABS-KEY(severe) AND TITLE-ABS-KEY(robotic) AND (TITLE-ABS-KEY("Action Research Arm Test") OR TITLE-ABS-KEY(ARAT) OR TITLE-ABS-KEY("Fugl-Meyer Assessment") OR TITLE-ABS-KEY(FMA)) AND (TITLE-ABS-KEY("randomized controlled trial") OR TITLE-ABS-KEY("randomized clinical trial")) AND (TITLE-ABS-KEY("upper limb") OR TITLE-ABS-KEY(hand))	27
	#3	TITLE-ABS-KEY(stroke) AND TITLE-ABS-KEY(severe) AND (TITLE-ABS-KEY(“brain-computer interface”) OR TITLE-ABS-KEY(“brain-machine interface”)) AND (TITLE-ABS-KEY("Action Research Arm Test") OR TITLE-ABS-KEY(ARAT) OR TITLE-ABS-KEY("Fugl-Meyer Assessment") OR TITLE-ABS-KEY(FMA)) AND (TITLE-ABS-KEY("randomized controlled trial") OR TITLE-ABS-KEY("randomized clinical trial")) AND (TITLE-ABS-KEY("upper limb") OR TITLE-ABS-KEY(hand))	10
	#4	TITLE-ABS-KEY(stroke) AND TITLE-ABS-KEY(severe) AND (TITLE-ABS-KEY(“functional electrical stimulation”) OR TITLE-ABS-KEY(“neuromuscular electrical stimulation”)) AND (TITLE-ABS-KEY("Action Research Arm Test") OR TITLE-ABS-KEY(ARAT) OR TITLE-ABS-KEY("Fugl-Meyer Assessment") OR TITLE-ABS-KEY(FMA)) AND (TITLE-ABS-KEY("randomized controlled trial") OR TITLE-ABS-KEY("randomized clinical trial")) AND (TITLE-ABS-KEY("upper limb") OR TITLE-ABS-KEY(hand))	12
	#5	TITLE-ABS-KEY(stroke) AND TITLE-ABS-KEY(severe) AND TITLE-ABS-KEY(“brain stimulation”) AND (TITLE-ABS-KEY("Action Research Arm Test") OR TITLE-ABS-KEY(ARAT) OR TITLE-ABS-KEY("Fugl-Meyer Assessment") OR TITLE-ABS-KEY(FMA)) AND (TITLE-ABS-KEY("randomized controlled trial") OR TITLE-ABS-KEY("randomized clinical trial")) AND (TITLE-ABS-KEY("upper limb") OR TITLE-ABS-KEY(hand))	6
	#6	TITLE-ABS-KEY(stroke) AND TITLE-ABS-KEY(severe) AND TITLE-ABS-KEY("virtual reality") AND (TITLE-ABS-KEY("Action Research Arm Test") OR TITLE-ABS-KEY(ARAT) OR TITLE-ABS-KEY("Fugl-Meyer Assessment") OR TITLE-ABS-KEY(FMA)) AND (TITLE-ABS-KEY("randomized controlled trial") OR TITLE-ABS-KEY("randomized clinical trial")) AND (TITLE-ABS-KEY("upper limb") OR TITLE-ABS-KEY(hand))	4
	#7	(TITLE-ABS-KEY(stroke) AND TITLE-ABS-KEY(severe) AND TITLE-ABS-KEY(“sensor-based training”) AND (TITLE-ABS-KEY("Action Research Arm Test") OR TITLE-ABS-KEY(ARAT) OR TITLE-ABS-KEY("Fugl-Meyer Assessment") OR TITLE-ABS-KEY(FMA)) AND (TITLE-ABS-KEY("randomized controlled trial") OR TITLE-ABS-KEY("randomized clinical trial")) AND (TITLE-ABS-KEY("upper limb") OR TITLE-ABS-KEY(hand)))	0
	#8	(TITLE-ABS-KEY(stroke) AND TITLE-ABS-KEY(severe) AND TITLE-ABS-KEY(tablet) AND (TITLE-ABS-KEY("Action Research Arm Test") OR TITLE-ABS-KEY(ARAT) OR TITLE-ABS-KEY("Fugl-Meyer Assessment") OR TITLE-ABS-KEY(FMA)) AND (TITLE-ABS-KEY("randomized controlled trial") OR TITLE-ABS-KEY("randomized clinical trial")) AND (TITLE-ABS-KEY("upper limb") OR TITLE-ABS-KEY(hand)))	0
	#9	TITLE-ABS-KEY(stroke) AND TITLE-ABS-KEY(severe) AND TITLE-ABS-KEY(“PC training”) AND (TITLE-ABS-KEY("Action Research Arm Test") OR TITLE-ABS-KEY(ARAT) OR TITLE-ABS-KEY("Fugl-Meyer Assessment") OR TITLE-ABS-KEY(FMA)) AND (TITLE-	0

		ABS-KEY("randomized controlled trial") OR TITLE-ABS-KEY("randomized clinical trial")) AND (TITLE-ABS-KEY("upper limb") OR TITLE-ABS-KEY(hand)))	
	#10	(TITLE-ABS-KEY(stroke) AND TITLE-ABS-KEY(severe) AND TITLE-ABS-KEY(exoskeleton) AND (TITLE-ABS-KEY("Action Research Arm Test") OR TITLE-ABS-KEY(ARAT) OR TITLE-ABS-KEY("Fugl-Meyer Assessment") OR TITLE-ABS-KEY(FMA)) AND (TITLE-ABS-KEY("randomized controlled trial") OR TITLE-ABS-KEY("randomized clinical trial")) AND (TITLE-ABS-KEY("upper limb") OR TITLE-ABS-KEY(hand)))	3
	#11	(TITLE-ABS-KEY(stroke) AND TITLE-ABS-KEY(severe) AND TITLE-ABS-KEY(technology) AND (TITLE-ABS-KEY("Action Research Arm Test") OR TITLE-ABS-KEY(ARAT) OR TITLE-ABS-KEY("Fugl-Meyer Assessment") OR TITLE-ABS-KEY(FMA)) AND (TITLE-ABS-KEY("randomized controlled trial") OR TITLE-ABS-KEY("randomized clinical trial")) AND (TITLE-ABS-KEY("upper limb") OR TITLE-ABS-KEY(hand)))	5
	#12	(TITLE-ABS-KEY(stroke) AND TITLE-ABS-KEY(severe) AND TITLE-ABS-KEY("serious game") AND (TITLE-ABS-KEY("Action Research Arm Test") OR TITLE-ABS-KEY(ARAT) OR TITLE-ABS-KEY("Fugl-Meyer Assessment") OR TITLE-ABS-KEY(FMA)) AND (TITLE-ABS-KEY("randomized controlled trial") OR TITLE-ABS-KEY("randomized clinical trial")) AND (TITLE-ABS-KEY("upper limb") OR TITLE-ABS-KEY(hand)))	1
PubMed	#1	stroke[Title/Abstract]) AND (severe[Title/Abstract]) AND (rehabilitation[Title/Abstract]) AND (("Action Research Arm Test"[Title/Abstract]) OR (ARAT[Title/Abstract]) OR ("Fugl-Meyer Assessment"[Title/Abstract]) OR (FMA[Title/Abstract])) AND (("randomized controlled trial"[Title/Abstract]) OR ("randomized clinical trial"[Title/Abstract])) AND (("upper limb"[Title/Abstract]) OR (hand[Title/Abstract]))	18
	#2	stroke[Title/Abstract]) AND (severe[Title/Abstract]) AND (robotic[Title/Abstract]) AND (("Action Research Arm Test"[Title/Abstract]) OR (ARAT[Title/Abstract]) OR ("Fugl-Meyer Assessment"[Title/Abstract]) OR (FMA[Title/Abstract])) AND (("randomized controlled trial"[Title/Abstract]) OR ("randomized clinical trial"[Title/Abstract])) AND (("upper limb"[Title/Abstract]) OR (hand[Title/Abstract]))	6
	#3	stroke[Title/Abstract]) AND (severe[Title/Abstract]) AND ((“brain-computer interface”[Title/Abstract]) OR (“brain-machine interface”[Title/Abstract])) AND (("Action Research Arm Test"[Title/Abstract]) OR (ARAT[Title/Abstract]) OR ("Fugl-Meyer Assessment"[Title/Abstract]) OR (FMA[Title/Abstract])) AND (("randomized controlled trial"[Title/Abstract]) OR ("randomized clinical trial"[Title/Abstract])) AND (("upper limb"[Title/Abstract]) OR (hand[Title/Abstract]))	1
	#4	stroke[Title/Abstract]) AND (severe[Title/Abstract]) AND ((“functional electrical stimulation”[Title/Abstract]) OR (“neuromuscular electrical stimulation”[Title/Abstract])) AND (("Action Research Arm Test"[Title/Abstract]) OR (ARAT[Title/Abstract]) OR ("Fugl-Meyer Assessment"[Title/Abstract]) OR (FMA[Title/Abstract])) AND (("randomized controlled trial"[Title/Abstract]) OR ("randomized clinical trial"[Title/Abstract])) AND (("upper limb"[Title/Abstract]) OR (hand[Title/Abstract]))	2
	#5	stroke[Title/Abstract]) AND (severe[Title/Abstract]) AND (“brain stimulation”[Title/Abstract]) AND (("Action Research Arm Test"[Title/Abstract]) OR (ARAT[Title/Abstract]) OR ("Fugl-Meyer Assessment"[Title/Abstract]) OR (FMA[Title/Abstract])) AND (("randomized controlled trial"[Title/Abstract]) OR ("randomized clinical trial"[Title/Abstract])) AND (("upper limb"[Title/Abstract]) OR (hand[Title/Abstract]))	1
	#6	stroke[Title/Abstract]) AND (severe[Title/Abstract]) AND (“virtual reality”[Title/Abstract]) AND (("Action Research Arm Test"[Title/Abstract]) OR (ARAT[Title/Abstract]) OR ("Fugl-Meyer Assessment"[Title/Abstract]) OR (FMA[Title/Abstract])) AND (("randomized controlled trial"[Title/Abstract]) OR ("randomized clinical trial"[Title/Abstract])) AND (("upper limb"[Title/Abstract]) OR (hand[Title/Abstract]))	1

#7	stroke[Title/Abstract]) AND (severe[Title/Abstract]) AND ("sensor-based training"[Title/Abstract]) AND (("Action Research Arm Test"[Title/Abstract]) OR (ARAT[Title/Abstract]) OR ("Fugl-Meyer Assessment"[Title/Abstract]) OR (FMA[Title/Abstract])) AND (("randomized controlled trial"[Title/Abstract]) OR ("randomized clinical trial"[Title/Abstract])) AND (("upper limb"[Title/Abstract]) OR (hand[Title/Abstract]))	0
#8	stroke[Title/Abstract]) AND (severe[Title/Abstract]) AND (tablet[Title/Abstract]) AND (("Action Research Arm Test"[Title/Abstract]) OR (ARAT[Title/Abstract]) OR ("Fugl-Meyer Assessment"[Title/Abstract]) OR (FMA[Title/Abstract])) AND (("randomized controlled trial"[Title/Abstract]) OR ("randomized clinical trial"[Title/Abstract])) AND (("upper limb"[Title/Abstract]) OR (hand[Title/Abstract]))	0
#9	stroke[Title/Abstract]) AND (severe[Title/Abstract]) AND ("PC training"[Title/Abstract]) AND (("Action Research Arm Test"[Title/Abstract]) OR (ARAT[Title/Abstract]) OR ("Fugl-Meyer Assessment"[Title/Abstract]) OR (FMA[Title/Abstract])) AND (("randomized controlled trial"[Title/Abstract]) OR ("randomized clinical trial"[Title/Abstract])) AND (("upper limb"[Title/Abstract]) OR (hand[Title/Abstract]))	0
#10	stroke[Title/Abstract]) AND (severe[Title/Abstract]) AND (exoskeleton[Title/Abstract]) AND (("Action Research Arm Test"[Title/Abstract]) OR (ARAT[Title/Abstract]) OR ("Fugl-Meyer Assessment"[Title/Abstract]) OR (FMA[Title/Abstract])) AND (("randomized controlled trial"[Title/Abstract]) OR ("randomized clinical trial"[Title/Abstract])) AND (("upper limb"[Title/Abstract]) OR (hand[Title/Abstract]))	0
#11	stroke[Title/Abstract]) AND (severe[Title/Abstract]) AND (technology[Title/Abstract]) AND (("Action Research Arm Test"[Title/Abstract]) OR (ARAT[Title/Abstract]) OR ("Fugl-Meyer Assessment"[Title/Abstract]) OR (FMA[Title/Abstract])) AND (("randomized controlled trial"[Title/Abstract]) OR ("randomized clinical trial"[Title/Abstract])) AND (("upper limb"[Title/Abstract]) OR (hand[Title/Abstract]))	1
#12	stroke[Title/Abstract]) AND (severe[Title/Abstract]) AND ("serious game"[Title/Abstract]) AND (("Action Research Arm Test"[Title/Abstract]) OR (ARAT[Title/Abstract]) OR ("Fugl-Meyer Assessment"[Title/Abstract]) OR (FMA[Title/Abstract])) AND (("randomized controlled trial"[Title/Abstract]) OR ("randomized clinical trial"[Title/Abstract])) AND (("upper limb"[Title/Abstract]) OR (hand[Title/Abstract]))	0

Table S2 – Experimental and control intervention details of the studies included in the review. For each study the following data are reported: name of first author and publication year, intervention type, comparator and details about interventions.

First Author Name, Year of Publication	Intervention Type	Comparator	Details
Hesse, 2008	PS, Robotic	Different Technology	The study compares mechanical arm training with an electromechanical device versus FES, both in add-on to conventional therapy.
Lo, 2010	Robotic	Dose Equivalent UL training, Usual Care	The control group undergoing intensive comparison treatment was administered a structured protocol using conventional rehabilitative techniques, such as assisted stretching, shoulder-stabilization activities, arm exercises, and functional reaching tasks (equal doses with respect to robotic treatment). A further control group received customary care available to all patients.
Weber, 2010	PS	Without Technology	The study investigates FES combined with botulinum injection and home exercise program versus botulinum injection and home exercise program (without FES).
Shindo, 2011	PS	Without technology	The experimental group used an integrated volitional electrical stimulator with a splint while the control group used a traditional splint device. Both groups were instructed to use their affected hand as much as possible while wearing the devices in the activities of daily living.
Rosewilliam, 2012	PS	Usual Care	Both groups received a defined module of upper limb physiotherapy that reflected current local clinical practice in addition to the routine treatment (the experimental group only received neuromuscular stimulation).
Ochi, 2013	NIBS, Robotic	Different Parameters	Anodal tDCS on the affected hemisphere was compared to cathodal tDCS on the unaffected hemisphere, both in addition to robot-assisted arm training.
Cordo, 2013	PS, Robotic	Different Technology	Robot assisted movement and muscle vibration were combined with EMG biofeedback in one study group and with torque feedback in the other.
Krewer, 2014	PS	Sham Stimulation	Repetitive peripheral magnetic stimulation was delivered with a dedicated inactive coil for sham stimulation.
Au-Yeung, 2014	PS	Sham Stimulation, Usual Care	Sham peripheral stimulation in the control group was delivered with a device in which the electrical circuits had been disconnected internally, meaning that the machines' lights and dial appeared to work normally when they were switched on. A further control group received usual care.
Ang, 2015	BCI, Robotic	Different Combination	In the target group, MI based BCI training was combined with online feedback delivered via Manus robot; in the control group, therapy with the Manus robot was delivered alone.
Pichiorri, 2015	BCI, VR and Visual	Without Technology	The control group performed MI training with no BCI assistance: the patients were instructed to imagine the same movements as in the BCI-based MI training (grasping and finger extension) with their affected hand.
Pennati, 2015	Robotic	Different Combination	Robotic training alone was compared with robotic training plus botulinum injection.

First Author Name, Year of Publication	Intervention Type	Comparator	Details
Carrico, 2016	PS	Sham Stimulation	Fort sham peripheral nerve stimulation, stimulus intensity was set to zero.
Wu, 2016	Robotic	Usual Care	Robot assisted therapy was delivered with InMotion robot and compared to an intensive upper limb training supervised by the same therapist. A further control group receive usual care treatment which was not dictated by the study protocol, the activities of this group were not recorded.
Frolov, 2017	BCI, Robotic	Sham Control	Sham BCI control performed with random feedback delivery (exoskeleton feedback unrelated to MI-induced EEG modulations)
Tomic, 2017	Robotic	Dose Equivalent UL Training	Matched Arm Training consisted of occupational therapy similar in structure and amount to the Robotic Training
Schick, 2017	PS	Different Combination	The intervention group received EMG triggered electrical stimulation combined with mirror therapy, while the control group received EMG triggered electrical stimulation alone.
Brunner, 2017	VR and Visual	Dose Equivalent UL Training	The control group received additional matched conventional training focused on task-related practice, in equivalent dose as VR-based training.
Rabadi, 2017	NIBS	Sham Stimulation	For sham tDCS stimulation, the device was switched off after the initial 30 seconds, out of the field of view of the patient.
Marquez-Chin, 2017	PS	Usual Care	FES was compared with conventional therapy that consisted of muscle facilitation exercises, task-specific repetitive functional training, stretching exercises, electrical stimulation for muscle strengthening (not functional training or FES therapy), activities of daily living, and caregiver training.
Carrico, 2018	PS	Sham Stimulation	For sham nerve stimulation, stimulator amplitude was set to zero after threshold identification.
Ding, 2018	VR and Visual	Dose Equivalent UL Training	The control group received equivalent training with a therapist consisting in the same exercises and number of repetitions as in the experimental group (camera-based mirror visual feedback).
Conroy, 2019	Robotic	Different Combination	Robot assisted therapy combined with therapist assisted task specific training was confronted with robot assisted therapy alone (control group).
Rodgers, 2019	Robotic	Dose Equivalent UL Training, Usual care	The control group received dose equivalent (with respect to robotic group) enhanced upper limb training, consisting in goal-oriented repetitive functional task practice. A further control group received usual care only.
Ramos-Murguialday, 2019	BCI, Robotic	Sham Control	Sham BCI control was implemented by delivering the feedback randomly, i.e., movement of the orthosis were unrelated to patients' EEG changes.
Takebayashi, 2020	Robotic	Dose Equivalent UL Training	The control group underwent dose equivalent (with respect to robotic group) self-guided upper limb training based on a set of personalized exercises established by a therapist.
Lee, 2020	Robotic	Different Technology	The study compares an end effector robot (InMotion2) with an exoskeleton (Armeo Power).
Chew, 2020	BCI, NIBS	Sham Stimulation	Sham tDCS Stimulation was delivered with similar parameters as in the target group but the stimulator was switched off after 20 seconds.

First Author Name, Year of Publication	Intervention Type	Comparator	Details
Lin, 2021	VR and Visual	Without technology	The control group underwent traditional mirror therapy (in contrast to the experimental group in which mirror therapy was delivered through VR goggles).
Hu, 2021	BCI, VR and Visual	Without technology	MI in the control group consisted in both simple joint movements (proximal and distal) and goal-oriented tasks. The intervention in study is a motor imagery based BCI with multimodal feedback.
Boasquevisque, 2021	NIBS	Sham Stimulation	Sham tDCS stimulation was delivered for 30 s including the ramping and then switched off for the remaining of the session.
Dawson, 2021	PS	Sham Stimulation	All patients were implanted with the vagus nerve stimulation device; all patients received five stimulations in reducing intensities at the beginning of each session, and then real or sham stimulation according to group allocation for the remaining of the session.
Llorens, 2021	NIBS, VR and Visual	Usual Care	The control group underwent conventional physical therapy alone. The intervention in study was tDCS combined with VR-based therapy.
Cantillo-Negrete, 2021	BCI, Robotic	Usual Care	The control group underwent conventional physical therapy alone. The intervention in study was a MI-based BCI with robotic hand orthosis feedback.
Takebayashi, 2022	Robotic	Different parameters	Different levels of assistance of the ReoGo system were compared.
Jiang, 2022	PS	Usual Care	The conventional physiotherapy included one session per day of conventional physical therapy, occupational therapy and neuromuscular electrical stimulation.
Schrader, 2022	Robotic	Without technology	The control group underwent traditional mirror therapy while in the experimental group the affected hand was also moved passively by the robotic glove during mirror therapy.
Ohnishi, 2022	PS	Different parameters, Usual Care	Suprathreshold NMES was confronted with repetitive facilitative exercises of the upper limb and with subthreshold + repetitive facilitative exercises. A further control group underwent usual care alone.
Huang, 2022	NIBS	Sham Stimulation	Sham tDCS stimulation was provided by a 10-s ramp up/down to the desired current intensity at the beginning and at the end of the session, with other no current delivered during the session
Wong, 2022	PS	Without technology	Unilateral task-oriented training delivered in both groups consisted in gross motor training, fine motor training, strength training, and activities of daily living training. The HAND orthosis (intervention in study) is an integrated volitional electrical stimulator with a splint.
Cordo, 2022	PS, Robotic	Different parameters	Control group participants were instructed to keep the affected UL as relaxed as possible (i.e., as opposed to active assistance in the experimental group), received vibration at lower frequency with respect to the experimental group and watched photographs depicting attractive landscapes instead of volitional torque biofeedback.

First Author Name, Year of Publication	Intervention Type	Comparator	Details
Takebayashi, 2022	Robotic	Without technology	Robotic self-training plus occupational therapy was confronted with robotic self-training plus CIMT, a further control group performed self-training (sanding, placing, stretching, and repetitive reaching/grasping/ releasing practice targeting the shoulder, elbow, and forearm of the paretic upper extremity) and occupational therapy.
Williamson, 2023	NIBS	Different Parameters, Sham Stimulation	The employed HD-tDCS unit has an automatic sham feature, which produces a sham waveform based on the indicated "real" waveform by only ramping the current to 2 mA at the start and end of the stimulation to provide the same feeling as active stimulation to the participants.
Dawson, 2023	PS	Sham Stimulation	All patients were implanted with the vagus nerve stimulation device; all patients received five stimulations in reducing intensities at the beginning of each session, and then real or sham stimulation according to group allocation for the remaining of the session.
Wang, 2023	NIBS, Robotic	Different Technology, Usual care	Robotic therapy with a soft robotic glove and rTMS were compared with conventional treatment in the control group (physical therapy, acupuncture, and occupational therapy)
Chen, 2023	Robotic	Usual Care	The experimental group treated with the Armule was compared to a control group receiving conventional therapy (including motomed-driven passive range of motion, manual assisted/active/resistive movement training, and repetitive functional task practice).
Feingold-Polak, 2024	Robotic	Different Technology, Usual Care	Exercises were guided by a Socially Assistive Robot in the experimental group and by a conventional personal computer in the control group. A further control group underwent usual care alone.
Brunner, 2024	BCI, PS	Usual Care	Patients in the control group received standard physiotherapy and occupational therapy according to clinical guidelines during the same period. For patients with severe UL paresis the methods of choice comprised mirror therapy, passive movements, electrical and sensory stimulation.

Legend:
BCI: Brain-Computer Interface; CIMT: Constraint Induced Movement Therapy; EEG: electroencephalography; EMG: electromyography; FES: Functional Electrical Stimulation; HD-tdCS: High-Definition transcranial direct stimulation; MI: motor imagery; NIBS: Non-Invasive Brain Stimulation; NMES: Neuromuscular Electrical Stimulation; PS: Peripheral Stimulation; rTMS: repetitive Transcranial Magnetic Stimulation; UL: upper limb; tDCS: transcranial Direct Current Stimulation; VR: Virtual Reality.
